# Effects of the high-inequality of income on the breast cancer mortality in Brazil

**DOI:** 10.1038/s41598-019-41012-8

**Published:** 2019-03-12

**Authors:** Francisco Winter dos Santos Figueiredo, Fernando Adami

**Affiliations:** Epidemiology and Data Analysis Laboratory, Faculdade de Medicina do ABC, Santo André, São Paulo Brazil

## Abstract

As well as breast cancer mortality, the income inequality in Brazil is different between Federative units, including between units of the same region. To assess the effects of the high-inequality of income on breast cancer mortality in Brazilian Federative Units, in the 2010 year. This is an ecologic study. Deaths from breast cancer in Brazilian women according to Federative units were obtained from the Department of Informatics of the Unified Health System. Mortality by breast cancer was estimated per 100,000 women and age-standardized by the direct method according to World Health Organization population. Income inequality was measured by the Gini index obtained from the United Nations Development Programme. The High-inequality of income was classified by the third tercile of the distribution from the Gini index of the Federative units. Univariate analysis was performed according to data normality. Linear regressions were performed by the stepwise backward method. The confidence level was 5%. Stata® (Stata Corp, LC) 11.0. was used. The High-inequality of income was associated with worse social and demographic indicators. The age-standardized breast cancer mortality was larger in the high-inequality of income Federative units. In the adjusted analysis, these Federative units presented a mean of 2 more deaths (ranging from the 0.4 to 3.7 deaths, r² = 0.79; p = 0.018) by breast cancer per 100,000 women when compared to the Federative units without high-inequality of income. In the Brazilian Federative units, the high-inequality of income was associated with age-standardized breast cancer mortality more.

## Introduction

Most countries have presented changes in indicators related to income inequality, a fact associated with the improvement of economic development^1^. However, this occurs both in developed countries such as the United States^2,3^ as well in developing countries such as Brazil^4^.

The decline in inequality is not unique to Brazil. Other Latin American countries like Argentina and Mexico, for example, had a similar fall due to different strategies. While in the two first countries the fall is attributed to the decrease in the remuneration of skilled labor, in Brazil, the lowest inequality of income is attributed to better strategies of the income distribution^5^.

There is an important relationship between income inequality and mortality from breast cancer in Brazil^6^, where breast cancer is one of the main causes of death among women^7^. If on the one hand, there was more investment of Brazilian public policies to prevent the burden of breast cancer^8^, on the other hand, there was a significant reduction in income inequality^6,9,10^.

However, this reduction in the income inequality did not happen equally for all Federative units Brazilians, that may have impacted for different health outcomes^11^. For example, some Brazilian Federative units in 2010 presented high indicators of income inequality such as the Amazonas (Gini Index of 0.65), Alagoas (Gini Index of 0.63), and Acre (Gini Index of 0.63), and others had lower income inequality, such as Santa Catarina (Gini Index of 0.49) Paraná (Gini Index of 0.54) and Mato Grosso (Gini Index of 0.55)^6^.

In this sense, having as a hypothesis that there is a direct relationship between the level of income inequality and the health of populations, we ask ourselves: there is a higher rate of breast cancer mortality in regions with high-inequality of income when compared to regions with low/moderate income inequality? The objective was to analyze the effects of high-inequality of income on mortality for breast cancer in Brazilian federal units in 2010.

## Results

**Figure 1:**
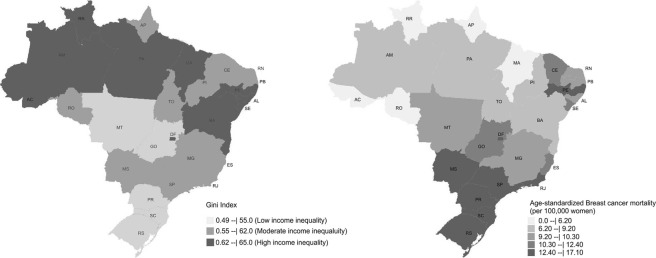
Distribution of Federative units and Brazilian Federal District according to levels of income-inequality and age-standardized mortality from breast cancer in 2010 (per 100,000 women). Acre (AC), Alagoas (AL), Amapá (AP), Amazonas (AM), Bahia (BA), Ceará (CE), Distrito Federal (DF), Espírito Santo (ES), Goiás (GO), Maranhão (MA), Mato Grosso (MT), Mato Grosso do Sul (MS), Minas Gerais (MG), Pará (PA), Paraíba (PB), Paraná (PR); Pernambuco (PE), Piauí (PI), Rio de Janeiro (RJ), Rio Grande do Norte (RN), Rio Grande do Sul (RS), Rondônia (RO), Roraima (RR), Santa Catarina (SC), São Paulo (SP), Sergipe (SE), Tocantins (TO).

In Fig. 1 we present the spatial distribution of income inequality - assessed by the Gini index -, and breast cancer mortality in Brazil in the 2010 year. The Federative units with high-inequality of income (Gini ≥ 0.62) are also the ones with the worst socioeconomic and development indicators.

This is reflected by the lower rate of aging (average difference of −1.5 (CI 95% −0.3; −2.8); p = 0.01), highest percentage of poverty (median difference of 18% more, ranging from 10.8 to 25.6%, p < 0.001), greater proportion of women under the age of 18 and who have children (average difference of 0.9, ranging from 0.3 to 1.6% more women per 100,000 women, p = 0.008) and lower Human Development Index for longevity (average difference of −0.03 (CI 95% −0.003; −0.05; p = 0.02)) and Human Development Index to education (average difference of −0.05 (CI 95% −0.1; −0.01); p = 0.02) found in the Federative units where high-inequality of income is present (Table 2).

Based on these differences, we analyzed the impact of high-inequality of income on breast cancer mortality. In model 1, which analyzed the mean difference in age-standardized breast cancer mortality according to the high-inequality of income adjusted for income per capita, no statistically significant differences were observed (mean difference of −1.5 (CI 95% −3.8; 1.5); r² = 0.38; p = 0.001).

**Figure 2:**
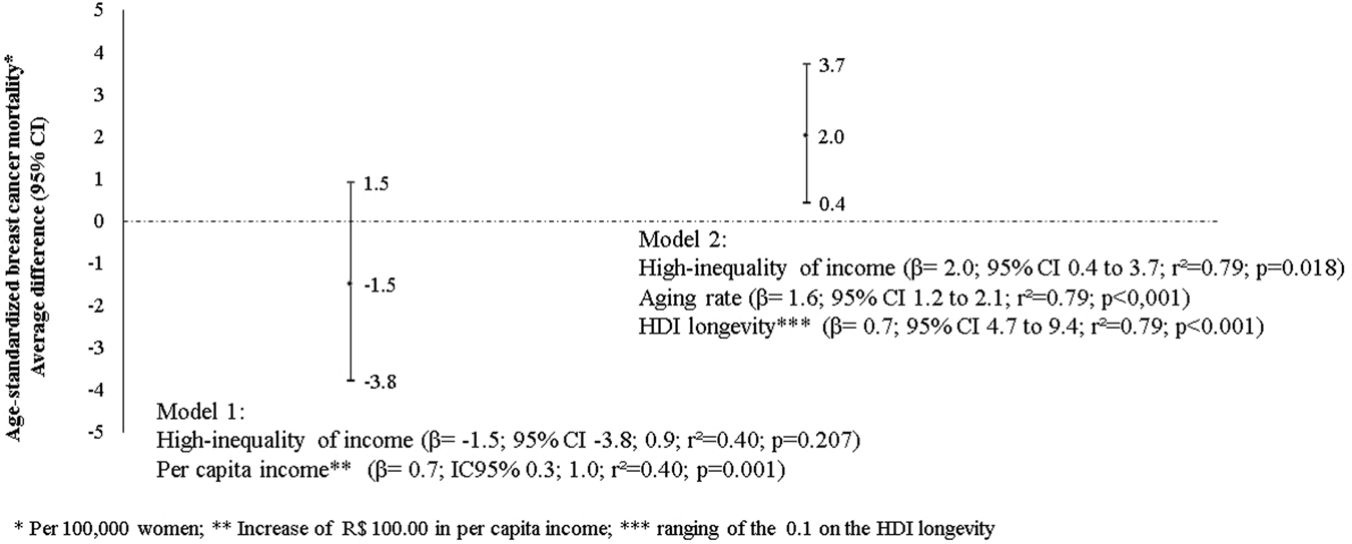
Models explaining the average difference of the age-standardized breast cancer mortality (per 100,000 women) according to Federative units with high-inequality of income compared to Federative units with low/moderate inequality of income in 2010.

On the other hand, when we adjusted the age-standardized breast cancer mortality by aging index and HDI longevity (model 2) – variables present after exclusion in the statistical model -, we observed that Federative units with high-inequality of income show 2 more deaths (ranging to 0.4 to 3.7 death per 100,000 women, r² = 0.79; p < 0.001) by breast cancer when compared to Federative units with low/moderate income inequality (Fig. 2).

## Discussion

Analyzing the differences in mortality rates for breast cancer among Federative units with high-inequality of income compared to Federative units with low/moderate inequality of income, we found that higher mortality due to breast cancer where there is a high-inequality of income.

However, the relationship between high-inequality of income and mortality due to breast cancer was observed after adjustment made by the Human Development Index and the aging index. The adjustment of the analyzes by confounding variables is one of the main steps necessary to understand the behavior of the variables before taking decisions to accept or reject hypotheses^12^ and is know that the breast cancer mortality is associated with the aging and with the Human Development Index^13^. As found in the present study, this change in the relationship between variables after adjustment was also observed in another study^14^ about the income inequality.

Additionally, the HDI is related to the mortality due to breast cancer such as the improving of the health services quality and consequently, to increasing of the life expectancy^15^. Another important issue is that in middle-high income countries, the relative income is a main associated factor to the health.

In Brazil, the great part of the population lives with the elderly and was dependent on these elderlies and your income. Thus, the increase in longevity can be associated with the reduction of income inequality in Brazil^15,16^. In this country, there are public policies related to increasing of income such as the improvement of the minimum wage, adjustment in the security benefits laws and Brazilian social protection programs such as *Bolsa família*^17^ to the population in general, but also policy directed to elderlies such as the retirement by age or by the fiscal contribution^16^.

The rapid demographic transition between 2001 and 2011 has increased income inequality, especially among the poorest^18^, such as inequality between regions^19^. On the other hand, the investment in health^20^ and the increase of the minimum wage^21^ were factors related to the decrease of the income inequality in the Federative units and federal district and that are related to better conditions of access to health in this country.

With high-inequality of income in Brazil, there are Federative units with lower economic power and few in the opposite situation. This can make it increase of cost of living of inhabitants and the need and of the state provide resources to the health. However, the resources and healthcare are unequal among Brazilian administrative regions, mainly for the breast cancer diagnosis and delay to the confirmation^22,23^. One of the main factors related to the increase in mortality is the late diagnosis of the cases, which makes treatment difficult and increases the lethality of the cases^23^.

Living in less developed regions and low educational level are factors that increase the risk of late diagnosis of breast cancer in Brazilian women^24^, a fact that also occurs in different regions of Europe^25^ and Hong Kong^26^. With less access to health services, lower education and unhealthy living habits, people are more likely to develop chronic diseases such as breast cancer and to have the diagnosis of these chronic diseases when they are already in higher stages. When those people living in areas of high inequality are diagnosed with breast cancer, they are already at higher stages^13^, and associated with delays in the provision of health services^22^, the lethality of the cases is higher and shorter survival^23^.

In the present study, we found that high-income inequality is related to worse socioeconomic and developmental indicators. This is because the increase in income inequality is directly related to social determinants that influence health such as smoking habits, alcoholism, and low educational levels^1,27,28^. The increase in these indicators points to a scenario of less self-care, as well as lower access to the primary health service^29^. With the greatest income inequality, there is also exacerbation of the social determinants that are related to health, mainly to the life habits and health care, who are consequences of the social stressor and lack of resources related to the income inequality. For these reasons, it is common to find studies that report an association between income inequality and outcomes related to social behavior^30^, suicide rate^4^ or breast cancer^6,8^.

In addition, in the last decade, increased investment in strategies for early diagnosis and treatment of breast cancer through public policies for decentralization of the single health system^8^ and increased life expectancy^17^ are also factors related to mortality and the reduction of income inequality, respectively. In a scenario of changes related to income inequality, it is important to understand that the change in income inequality may influence in a different way the current income of the populations^31^, and in this sense, this is a field of knowledge that still has much to be discovered. In addition, understanding how high inequality of income is related to other health outcomes can be an important tool to understand the real impact of this characteristic on the health of populations.

We do not understand the results found as if reducing income inequality would save lives. On the other hand, we find here that the places where there is high inequality of income are susceptible to worse socioeconomic conditions and consequently, lower conditions to have adequate health, which reflected in higher mortality due to breast cancer in the Brazilian Federative units with this characteristic in the 2010 year.

When we analyze the effects of high-inequality of income on breast cancer mortality in Brazilian Federative units in 2010, we found that there is higher mortality due to breast cancer in the Federative units, where there is a high-inequality of income. In this sense, it is necessary that there be adequate public policies for breast cancer for each reality at the state level, given the existing income inequalities, as well to understand the impact of high-inequality of income on the other health outcomes.

## Methods

### Study design

This is an ecologic study.

### Geographical and temporal delimitation

This study was performed at the 2018 year with 2010 year data. The analysis units were the Brazilian Federative Units and the choice of the year was because of the demographic sense performed in 2010.

### Data sources

They were used as data sources used for other epidemiological studies, whose reliability and validity has already been described^6,32^. Were used the database of the Department of Informatics of the Single Health System (Sistema Único de Saúde - DATASUS; www.datasus.gov.br), the Atlas Brazil, provided by the United Nations Development Program (UNDP, www.atlasbrasil.org.br/) and the database with information on the Federative units and Federal District of the Brazilian Institute of Geography and Statistics (IBGE, ibge.gov.br/estadosat/).

#### Variables

High-inequality of income – exposure variable: The Gini index from the Federative units was obtained from the Brazil Atlas. The Atlas was available by the United Nations Development Programme (UNDP). The income inequality was classified as low/moderate (first and second tertiles) and high (third tertile) according to the distribution endings of the Gini Index of Federative units and the Brazilian Federal District.

Thus, were considered as high-inequality of income the Federative units with Gini index higher than 0.62 and as low/moderate inequality of income states with values less than or equal to 0.62.

Mortality from breast cancer - outcome variable: The deaths owing to breast cancer were obtained from the Department of Informatics of the Unified Health System (DATASUS) through the Mortality Information System (*Sistema de Informações sobre Mortalidade*, SIM), defined according to the Tenth International Classification of Diseases (ICD-10)^33^.

The population of women was obtained from the Brazilian Institute of Geography and Statistics (IBGE) using data from the 2010 Demographic Census. Crude mortality was calculated per 100,000 women. Crude rates were standardized by age using the direct method, using the standard population of the World Health Organization (WHO)^34^.

Sociodemographic characteristics - adjustment variables: The sociodemographic variables of the population according to Brazilian Federative units were obtained from the IBGE states, UNDP and DATASUS. The variables were presented in Table 1.

**Table 1:** Sociodemographic variables.

Variables	Source
Aging Index*	IBGE states (ibge.gov.br/estadosat/)
Per capita Income	IBGE states (ibge.gov.br/estadosat/)
Proportion of people living in poverty	IBGE states (ibge.gov.br/estadosat/)
Proportion of 25-year-old women with full tertiary education	IBGE states (ibge.gov.br/estadosat/)
Proportion of women under the age of 18 that have children	IBGE states (ibge.gov.br/estadosat/)
Human Development Index by income, longevity, and education	UNDP (http://www.br.undp.org/)
SIM coverage**	DATASUS (http://tabnet.datasus.gov.br)

*Number of people aged 65 or over 100 people per people with age 14 people.

**SIM coverage was estimated by the ratio between the number of deaths from defined causes reported in the SIM and the number of deaths identified by active search in the death certificates in registries.

**Table 2:** Sociodemographic characteristics of Federative units with high-inequality of income (Gini ≥ 0.62) compared to Federative units with low/moderate inequality of income (Gini < 0.62) in 2010.

Sociodemographic characteristics	Low/moderate inequality of income (Gini < 0.62)	High-inequality of income (Gini ≥ 0.62)	Difference	p-value
Aging (average)	7.0 (6.2; 7.8)	5.4 (4.5; 6.4)	−1.5 (−2.8; −0.3)	0.01*
% Poverty (median)	10.5 (7.2; 23.8)	29.1 (26.8; 33.7)	18 (10.8; 25.6)	<0.001**
SIM coverage (average)	87.5 (83.7; 91.3)	81.9 (74.4; 89.5)	5.6 (−1.5; 12.7)	0.12*
Income per capita (average)	744.1 (637.2; 851.0)	616.8 (582.6; 811.4)	127.3 (−109.1; 363.7)	0.28*
Women with 25 years of complete high school (median)	10.6 (18.3; 12.0)	8.1 (6.3; 9.8)	−2.3 (−4.9; 0.24)	0.07**
Women < 18 years old with children (average)	3.0 (2.6; 3.3)	3.9 (3.2; 4.7)	0.9 (0.3; 1.6)	0.008*
**Human Development Index**				
Income (average)	0.72 (0.70; 0.75)	0.68 (0.63; 0.73)	0.04 (−0.09; 0.005)	0.08*
Longevity (average)	0.82 (0.81; 0.83)	0.79 (0.77; 0.82)	−0.03 (−0.05; −0.003)	0.02*
Education (average)	0.63 (0.61; 0.66)	0.58 (0.53 0.62)	−0.05 (−0.1; −0.01)	0.02*

SIM: Sistema de Informações sobre Mortalidade of Brazilian Health Ministry.

*Student’s t-test **Mann-Whitney.

### Statistical analysis

The Shapiro-Wilk test was used to assess the distribution of quantitative variables. For the variables with a normal distribution (Shapiro-Wilk, p ≥ 0.05) the T-test was used and for the variables without normal distribution (Shapiro-Wilk, p < 0.05), was used Mann-Whitney test.

Linear regression was used to analyze the association between high-inequality of income and mortality due to breast cancer. Two models were tested: I) high-inequality of income adjusted for per capita income and II) high-inequality of income adjusted by the other variables in the model after application of the stepwise backward strategy, with input selection criteria of 0.05 and withdrawal of the model of 0.10.

The significance level was 5%. The program used for the statistical analysis was Stata 11.0® (Stata Corp, L C). Tabwin version 3.0 was used to create the maps.

### Ethics committee approval

According to Resolution No. 466 of 12 December 2012 of Brazilian National Health Council, there is no need for the ethical assessment of the Research Ethics Committee because the data analyzed are public and of unrestricted access and use.

## Data Availability

The data that support the findings of this study are available from the corresponding author (FWSF), upon reasonable request.
